# Mathematical modeling of intracellular signaling pathways

**DOI:** 10.1186/1471-2202-7-S1-S10

**Published:** 2006-10-30

**Authors:** Edda Klipp, Wolfram Liebermeister

**Affiliations:** 1 Max Planck Institute for Molecular Genetics, Ihnestr. 73, 14195 Berlin, Germany

## Abstract

Dynamic modeling and simulation of signal transduction pathways is an important topic in systems biology and is obtaining growing attention from researchers with experimental or theoretical background. Here we review attempts to analyze and model specific signaling systems. We review the structure of recurrent building blocks of signaling pathways and their integration into more comprehensive models, which enables the understanding of complex cellular processes. The variety of mechanisms found and modeling techniques used are illustrated with models of different signaling pathways. Focusing on the close interplay between experimental investigation of pathways and the mathematical representations of cellular dynamics, we discuss challenges and perspectives that emerge in studies of signaling systems.

## Introduction

Signaling pathways enable cells to sense changes in their environment, to integrate external or internal signals, and to respond to them by changes in transcriptional activity, metabolism, or other regulatory measures. The proper functioning of these pathways is crucial for adaptation and survival under varying conditions, but also for differentiation and cell fate. In multicellular organisms, signaling pathways play an important role in development and oncogenesis, but also in neural plasticity. Signaling pathways frequently consist of ubiquitous building blocks, such as receptors, ERK or MAP kinase cascades, G proteins and small G proteins, and their design seems to be conserved throughout all kingdoms of life.

At first glance, signaling can be seen as a linear connection between input elements (the receptors) and output elements (such as regulators of gene expression). A closer inspection reveals that signaling pathways interact with each other, forming a network. Schwartz [1] introduced the notion of crosstalk, referring to the case that two inputs (here: growth factors from different families) work through distinct signaling pathways but cooperate to regulate cell growth. Intensive experimental work has revealed numerous potential paths for crosstalk.

In order to understand the complex behavior of signaling networks, researchers have adopted computational modeling approaches, ranging from abstract models that emphasize some key features of signaling pathways [2,3] to detailed models that describe the dynamics of specific pathways in specific organisms [e.g. [4-7]]. Several models from both categories have been developed and experimentally tested, which has revealed interesting behavior. Overviews on structural properties and dynamic features of signaling pathway models are given in [8,9].

Modeling of biochemical networks can help to integrate experimental knowledge into a coherent picture and to test, support, or falsify hypotheses about the underlying biological mechanisms. The behavior of complex systems is often hard to grasp by intuition, because our reasoning tends to follow simple causal chains: if feedback cycles come into play or if the relative timing of processes makes a difference, then mathematical simulation may be more reliable than mere intuition. Modeling emphasizes the holistic aspects of signaling networks, which disappear if the components are studied in separation in different wet labs around the globe. Furthermore, once a model has been established, it can be used to test hypotheses or simulate experiments that would be hard or impossible to do in the lab.

In this review, we will illustrate the modeling of signaling pathways with examples from different cell types and discuss some problems that we consider open in the modeling of such pathways. We will first present the building blocks of signaling pathways and describe the iterative process of model building and the integration of pathways. We will then outline mathematical techniques and general results that have been obtained by mathematical analysis. To illustrate these points, we will review a number of models that have been established for signaling systems in eukaryotic cells, with an emphasis on neuron cells.

## Components of signaling pathways

Despite their diversity in function and design, many signaling pathways use the same essential components, which are often highly conserved through evolution and between species. For example, proteins in yeast pathways have homologs in human pathways (e.g. Hog1 and p38, both MAP kinases that are active in osmoregulation). Here, we will introduce the most prevalent signaling pathway modules.

**Figure 1:**
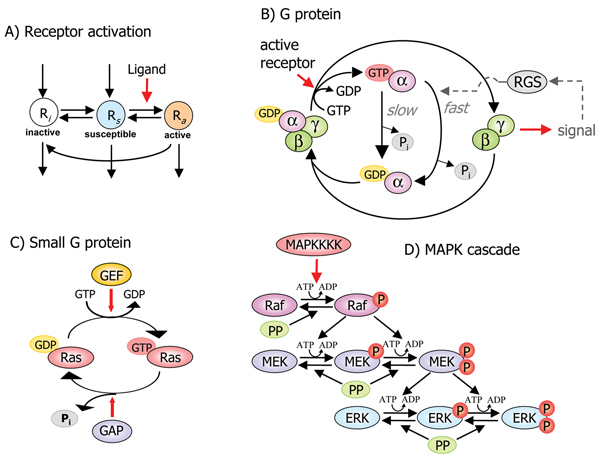
**Building blocks**. Building blocks or modules of signaling pathways. Despite their diversity, signaling pathways often employ a set of common components (modules, motifs, building blocks) including the depicted ones. A) Receptors sense specific ligands or stimuli and change their conformation from the susceptible form R_*s *_to the active form R_*a*_, which transfers the signal downstream. Cells can fine-tune their excitability by (i) changing the susceptibility of receptors, e.g. switch between R_*s *_and inactive form R_*i*_, or (ii) by regulating the number of receptors via production or internalization and degradation. B) G proteins are heterotrimers consisting of subunits α, β, and γ. In the ground state, the α-subunit is bound to GDP. The active receptor triggers the activation of the G protein by exchanging the GDP with a GTP. The G protein dissociates into its subunits, which transmit the signal downstream by binding to other proteins and activating or inhibiting biochemical processes. The α-subunit carries the GTP. After GTP hydrolysis, which is a highly regulated process on its own, the subunits can reassociate to form the initial heterotrimeric G protein. The Regulator of G protein signaling (RGS) is involved in a larger feedback loop. C) Small G proteins switch between GDP-bound or GTP-bound forms with different activities. Conversion from the GDP state to the GTP state is catalyzed by a so-called Guanine Exchange Factor (GEF), the reverse process is facilitated by a GTPase-activation protein (GAP), which induces hydrolysis of the bound GTP [13]. D) MAPK (mitogen activated kinase) cascades consist of three or four different proteins (the kinases) that specifically catalyze the phosphorylation of the subsequent kinases under consumption of ATP. In this case, Raf is a MAPKKK, MEK is a MAPKK, and ERK is a MAPK. The number of phosphorylation events on each level can differ. Dephosphorylation is exerted by phosphatases (denoted by PP).

Most **receptors **are transmembrane proteins that receive extracellular stimuli by ligand binding and transmit the signal to intracellular signaling molecules. Upon signal sensing, they dimerize or change their conformation and become active (Figure 1A), now being able to initiate downstream processes. Cells can regulate the number and the activity of specific receptors, e.g. in order to shut off the signal transmission during sustained stimulation. An interplay of production and degradation regulates the number of receptors (for a model involving receptor internalization in the yeast pheromone pathway see [6]). Phosphorylation of serine/threonine or tyrosine residues in the cytosolic domain by protein kinases can regulate the activity and thereby adapt the signaling system to input signals of different intensity. This tuning of sensors by feedback control can allow for exact adaptation as it is vital for bacterial chemotaxis[10,11].

Similarly, the calcium influx at AMPA-type glutamate receptors in glutamatergic synapses [12] can be tuned by the phosphorylation of serine residues. The next step in signaling is often the activation of **G proteins**. The heterotrimeric G protein consists of the subunits α, β, and γ (Figure 1B). Upon activation, a GDP bound to the α-subunit is exchanged with a GTP, and the G protein dissociates into different subunits which transmit the signal to downstream processes. As soon as the GTP is hydrolyzed to GDP, the subunits can reassociate to form the initial heterotrimeric G protein.

**Small G proteins **like Ras, Rho, Rab, Ran, or Arf are either bound to GDP or GTP and have different activities in both forms (Figure 1C). Transformation from the GDP state to the GTP state is catalyzed by the Guanine Exchange Factor (GEF), while the reverse process is facilitated by a GTPase-activating protein (GAP), which induces hydrolysis of the bound GTP [13].

Extracellular signal-regulated kinase **(ERK) **or mitogen-activated protein kinase **(MAPK) cascades **consist of three or four different proteins that specifically catalyze the phosphorylation of the subsequent proteins (Figure 1D). According to their roles, these kinases are called MAP kinase (MAPK), MAP kinase kinase (MAPKK), and so on. The dephosphorylation is ensured by phosphatases that are often less specific, but can also be very specific to certain targets. In some cases, the members of a signaling cascade form complexes with **scaffold proteins**. By binding the kinases, scaffolds can ensure the physical vicinity or even the correct molecular orientation. Scaffolding can account for the fact that signaling pathways often appear to be decoupled although they contain common components.

## Techniques in mathematical modeling of signaling pathways

### The purpose of modeling

Since a reliable quantitative model is hard to obtain, experimental knowledge is often first described with a raw model, which is then improved based on new experiments. In this way, model development and experimental design are improved in a synergistic manner. Frequently, both the model and the objective of the study are also iteratively refined (e.g. [14,15]). Although so far, no standard for the development of pathway models has been established, the majority of approaches (e.g. in [16-18]) embarks on the following general strategy: an initial model is constructed based on pre-existing data such as concentrations, kinetic parameters, flux measurements, microscopic images, which may stem from diverse sources (literature, databases, own measurements). First problems arise already at this stage: links can be missing or discrepancies between model outcome and biological observation can arise.  Resolving them requires new experimental data. The points that are not sufficiently defined in the network can also be analyzed in a computational approach that clarifies which of several alternative model assumptions may explain the observations. The initial model is then used to make predictions. These predictions are tested in new experiments or against new data for different mutant organisms or for different pathway stimulation scenarios. The results allow for a model update and a second round of iteration.

This point is nicely illustrated by the development of cell cycle models. First simple models were made to study the emergence of oscillations in cascades of post-translational modifications with feedback[19]. Since that time, dynamic models for cell cycle have been developed and iteratively improved [[20-22]20, 21, 22 and others], now comprising dozens of variables.

### Definition of the system

The most basic decision in model building concerns the model components: which molecules and interactions play a role and which of them will be left out? Omitting certain processes from the models is based on the assumption that they have only a minor influence on the event under study, that their values remain constant in the experimental setup, or that they simply cannot be described with the currently available means. For example, the effect of regulated gene expression is usually neglected in the modeling of metabolic networks although modelers are certainly aware of production and degradation of enzymes. But the different time scales of protein turnover and metabolic reactions justify this simplification in many cases.

On the contrary, the actual architecture of signaling pathways often depends on the cell state (developmental state, cell cycle, previous events). For example the pheromone receptor in yeast is degraded after stimulation; therefore the pathway now lacks its first element. Also other variables besides substance concentrations may play a role: Upon osmotic stress, the HOG pathway (see below) leads eventually to the production of glycerol and, thereby, to a regulation of cell volume and turgor pressure. This means that it essentially changes the state of the cell and, by means of the volume changes, the concentration of all involved components. This is a strong argument for including the volume changes into the model in order to understand the relevant regulatory interactions.

### Coupling of pathways

Different groups of researchers who are experts in their field have developed specific pathway models which appropriately describe the studied phenomena. In order to build larger networks, it is desirable to integrate those different models into larger networks that can reflect more or more complex biological phenomena – as it shall be exemplified below by Bhalla's models of neuronal signaling pathways.

To integrate pathways into larger models, the development of tools for a sensible integration of pathway models is clearly on the agenda. Important ingredients for coupling of models are (1) the development of standardized model exchange formats such as SBML [23], (2) the emergence of model databases such as JWS online [24], Biomodels  or the Database of Quantitative Cellular Signaling (DOQCS, ), and (3) quality standards for model description in publications, such as MIRIAM [25]. As an alternative, it has been proposed to construct signaling networks more or less automatically from data stored in databases, e.g. from interaction maps [26,27]. However, this approach is likely to miss important biological details: even experts for specific pathways sometimes start to stumble when they are asked for the correct sequence of events in the interaction of proteins – although it is well known that the proteins do interact, and which protein domains are involved.

### Mathematical structure of biochemical network models

Structural models describe the present molecules and their interactions and possibly the molecule numbers. If also the dynamics is to be considered, these numbers will change in time. To describe these changes, modelers can choose from different types of mathematical models. Models used for signaling pathways can be loosely grouped as follows: they can be (i) deterministic (with defined states in the future) or probabilistic (stochastic processes), (ii) discrete or continuous with respect to time and to component abundance (i.e. molecule numbers or concentrations), and they (iii) may or may not describe the processes in space. The choice of a model will depend on system, the available information, and the specific questions to be studied.

In most models, biochemical reaction systems are described in a deterministic, continuous manner by rate equations for the concentrations of substances and complexes. The mathematical representation is a set of ordinary differential equations (ODEs)



where *m *is the number of biochemical species with the concentrations *c*_*i*_, *r *is the number of reactions with the rates *v*_*j*_, and the quantities *n*_*ij *_denote the stoichiometric coefficients. Depending on experimental information, the individual reaction rates can be described by very sophisticated kinetic laws. But often, mass action kinetics is used, where the rate for the reaction  reads

*v *= A · B · *k*_*f *_- *C · k*_*b*_.

The parameters *k*_*f*_, *k*_*b *_are the rate constants. Especially in metabolic networks, the traditional Michaelis-Menten kinetics is used, where the rate for the enzyme-catalyzed reaction *S → P *is expressed as



The quantity *V*_*max *_is the maximal rate and *K*_*M *_denotes the substrate concentration ensuring a half-maximal rate.

In the deterministic framework, spatial distribution of compounds can be described by distinguishing different compartments or by describing dynamics in a continuous space with partial differential equations. Examples for systems that are discrete with respect to time and values of variables are Boolean networks [28], Petri nets [29], or cellular automata[30]. In systems with small molecule numbers, stochastic effects tend to become relevant, and individual reaction events have to be simulated, e.g., by the different algorithms put forward by Gillespie [31-33]. When the particle numbers are high, the results of stochastic simulations are often well approximated by deterministic rate equation models. There is, however, no easy way to decide in advance whether or not a deterministic description is justified.

### Signaling networks and metabolic networks

Modeling of biochemical reaction networks has gained much success in the field of metabolic pathways, and many techniques have been developed for studying metabolic systems (steady state analysis, MCA, stoichiometric analysis, independent fluxes, conservation relations, flux balance analysis etc.). Therefore, we may ask for the similarities and the differences between metabolic and signaling pathways, and whether techniques developed for metabolism also apply to signaling systems. Both metabolism and signaling are modeled by a set of biochemical reactions including binding, dissociation, complex formation, and transfer of molecule groups. Especially phosphorylation and dephosphorylation occur in both cases (e.g. phosphofructokinase in metabolism or MAP kinases in signaling). Nevertheless, we also encounter differences, such as the following:

(i) In metabolism, the amount of enzyme and substrate often differ by several orders of magnitude (concentrations in the order of nM compared to mM). This is a precondition for the application of Michaelis-Menten kinetics, which is only justified if the enzyme concentration is much lower than the substrate concentration (quasi-steady state assumption suggested by Briggs and Haldane, 1925 [34]). In signaling pathways, the numbers of catalyst and substrate molecules are usually in the same order of magnitude. For example, molecule numbers of the proteins involved in typical yeast signaling pathways vary between about several hundreds and several thousands. This is a strong argument for not applying the Michaelis-Menten approximation, but using mass action kinetics, at least as long as the detailed kinetics of that specific reaction is not known. Michaelis-Menten kinetics underestimate the reaction rate compared to (a) mass action kinetics for the whole reaction or (b) mass action kinetics for the individual steps including reversible binding and product release (which give about the same results). This may lead to qualitatively different behavior like the occurrence or absence of oscillations in a MAP kinase cascade with feedback.

(ii) While metabolic pathways are characterized by a flow of matter (an atom entering glycolysis at the upper end/hexokinase may leave it at the lower end/pyruvate kinase), signaling pathways comprise many closed loops in which matter flows, e.g., within the G protein cycle or between the different phosphorylation states of a protein. The essential function of signaling pathways is the flow of information, although this statement does not exclude that the flow of matter in metabolism is also connected to a flow of information.

(iii) Phosphorylation under consumption of nucleotide triphosphates (ATP) has a different function. While it serves as a fuel in metabolism (it essentially increases the difference in free energy) and speeds up the reactions, it just marks proteins as different (changes their activity or binding behavior) in signaling.

(iv) Although metabolism is able to respond to environmental changes, especially nutrition, it is mainly a homeostatic process and has a strong constant component. This is the basis for consideration of steady states in metabolism models. Metabolic control analysis [35,36], elementary flux modes [37], flux balance analysis [38] and other common approaches are based on the assumption of a steady state. Signaling pathways, on the opposite, operate essentially in a non-static manner: the pathway acts by state changes. Therefore, the analysis of their steady states cannot be central, although it may provide information on the contribution of different components to how such pathways are switched on or off, i.e. how they are shifted away from their resting state.

### Metabolic control analysis

Metabolic control analysis (MCA, [35,36,39], for an introduction see [40-42]) studies the effect of small parameter changes on concentrations and fluxes in the steady states of metabolic systems. The (linearized) influence of a certain parameter on a certain variable is quantified by response coefficients or control coefficients, which are also known as sensitivities. Metabolic control theory has developed the so-called summation and connectivity theorems, which relate the control coefficients to the stoichiometry and the linearized reaction kinetics of the system. Control coefficients can be used to detect important system parameters. This has been exemplified for a model of the WNT pathway [18], which we will discuss below. To quantify the robustness of a variable against general parameter changes, Lee et al [18] introduced a measure for robustness, ρ = 1/(1 + σ), where σ is the standard deviation of the concentration control coefficients. A high value of ρ implies robustness, or in other words, a low control of parameters over that variable. In recent years, MCA has been extended to cover time-dependent phenomena: Ingalls and Sauro [43] have proposed time-dependent response coefficients that describe the influence of parameter changes on time series. Besides this, control coefficients for signal characteristics such as maximal amplitude or mean signal time of output signals have been defined [44], and also spatial processes such as diffusion can be incorporated [45].

## Phenomena studied using mathematical models

Besides the modeling of specific biological pathways, mathematical models have been used to study general properties of signaling pathways. These studies have also revealed general design principles that relate the structure, the dynamics, and the function of pathways. Also simplified models do not only serve as first attempts when detailed information on the molecular mechanisms is missing; they may also help to emphasize and thus clarify essential features of the functioning of a system, even when abundant molecular information is available.

### Relative importance of kinases and phosphatases

The proper functioning of MAPK pathways depends on a balance of phosphorylation and dephosphorylation. Phosphatases counteract the effect of kinases, contribute to downregulation after pathway stimulation, and they also keep basal levels low despite activation by noise or sub-critical inputs. The ratio *k/p *between kinase activity (*k*) and phosphatase activity (*p*) determines both the basal level of active MAPK and the final level of MAPK upon continuous pathway stimulation. If the stimulation is transient, then *k *and *p *influence both amplitude and duration of the activation. Upon weak stimulation, *k *determines the amplitude and *p *the duration [46]. Upon strong stimulation these roles are changed, since mass conservation on each level of the kinase cascade prevents a stronger increase in the level of activated protein, which in turn results in prolonged activation [16].

### Signal amplification

It has been proposed that signaling cascades can amplify their signals, and it has been studied how this is actually achieved [2,47,48]**. **Amplification means that an input of a few signaling molecules can lead to a large number of active output molecules, which of course bears the danger that also noise is amplified. One way to quantify signal amplification is to compare the activated and the inactive state of a pathway. "Inactive" means that the input signal is shut off except for some inevitable noise. Amplification can then be defined as the relative increase of the output signal upon activation, divided by the relative increase of the input signal. Based on this definition, it was shown [16] for a simple model of MAP kinase cascades that amplification depends on the ratio *k*/*p *of the rate constants for kinase (*k*) and phosphatase (*p*) reactions. However, also the output level at the poorly activated state depends on *k*/*p*. Hence low *k*/*p *leads to high amplification at low absolute values, while high *k*/*p *leads to low amplification at high basal levels.

If the members of a MAP kinase cascade are bound to a scaffold protein, we would expect no amplification because every MAP kinase molecule will activate at most one MAP kinase molecule of the following level. However, a little modification of the scaffold mechanism can allow for amplification, as it is the case in the yeast pheromone pathway. Three MAP kinases are bound to a scaffold protein. If the phosphorylated MAPK Fus3 can dissociate from the complex allowing for a repeated binding, phosphorylation and release of further Fus3 molecules, then strong amplification can occur.

Also the numbers of protein molecules per cell, as given in the Yeast GFP Fusion Localization Database [49], suggest that a certain degree of signal amplification is envisaged in cellular signaling: their abundance increases from upper to lower parts of the pathways. For example, in yeast MAPK pathways the subsequent molecule numbers read: Ssk2+Ssk22: 274, Pbs2: 2160, Hog1: 6780 (Hog pathway) and Ste20: 259, Ste11: 736, Ste7: 672, Fus3: 8480 (pheromone pathway).

### Impact of feedback loops on regulatory properties

The role of feedback loops in signaling cascades has been discussed in detail in various publications (e.g. [9,50,51]). Feedback loops can alter the dynamical behavior of a signaling system and may accomplish different functions.

Negative feedback loops usually downregulate a pathway after successful transmission of the signal. In this way, the cell prevents sustained pathway activation in cases when the external signal is still present, but the internal adaptation machinery has already started (e.g. [16,52]). Depending on the kinetic parameters, negative feedback loops can also cause oscillations [51]. Positive feedback loops [17] may amplify the pathway activation such that small stimuli can result in pronounced effects. Depending on the kinetic design of the system they can also result in bistability, thereby ensuring that weak stimuli activate the pathway only transiently, while strong stimuli may result in a sustained activation.

### Crosstalk between pathways

The notion of crosstalk implies that signaling pathways can, to a good approximation, be regarded as almost isolated sets of reactions, which exchange only little information.

A number of studies are devoted to the crosstalk between different pathways [1] and also to the question of how pathways can avoid crosstalk although they share common protein species. In the context of modeling, it was analyzed how pathways using a common intermediate can pass different signals [53], and how signals from one pathway modulate the activity of another pathway [2]. As a quantitative measure of network crosstalk, the number of non-negative linear combinations of extreme pathways has been proposed in [3]. Extreme pathways are a concept from stoichiometric analysis and they describe a set of flux vectors that completely characterize the steady-state capabilities of the network. Another definition of crosstalk [54] refers to the relative frequency of cross-interactions, that is, the number of cross-interactions found divided by the maximal number of possible cross-interactions.

However, if one regards the multitude of signaling molecules and their possible interactions as one big signaling system ("signalosome", as put forward in [55]), then the communication among compounds is the rule and not the exception. From this point of view, the question of how to prevent or establish crosstalk is less fundamental and should be rephrased [56]: What is the biological function of the communication between parts of the signaling network?

## Models of signaling pathways in various cell types

To illustrate the concepts presented so far, we shall now present some signaling pathways that have been modeled quantitatively. According to their different functions, they show different designs, but they are mainly based on the same recurrent building blocks introduced above. We will not cover models for nerve excitation, neuronal firing or oscillations of the neuronal network as a whole and will also not refer to the integrate-and-fire model, the FitzHugh-Nagumo model or the Hodgkin-Huxley model. Instead, we concentrate on models of intracellular pathways that transmit their signals via protein-protein interactions or interactions with second messengers.

### Stress response pathways in yeast

**Figure 2:**
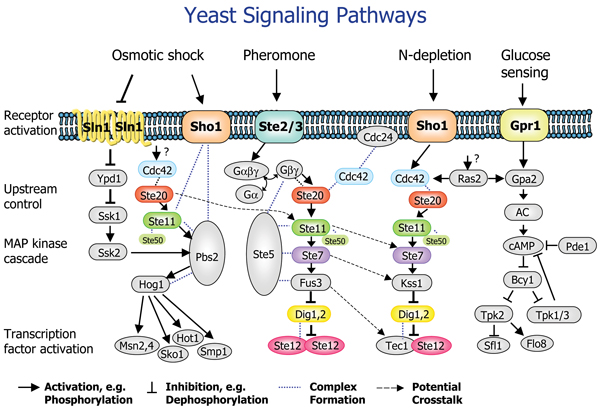
**Signaling Pathways in Yeast**. Overview of signaling pathways in the baker's yeast *S. cerevisiae*. From left to right: HOG pathway activated by osmotic shock, pheromone pathway activated by pheromones secreted by cells of the opposite mating type, pseudohyphal growth pathway stimulated by starvation conditions, and the glucose sensing pathway. In each pathway, the activated receptor activates a cascade of intracellular processes including complex formation, phosphorylation and dephosphorylation, and transport steps. Most of these pathways comprise a MAP kinase cascade and transcription factors that regulate the expression of target genes. Besides this, the signaling pathways can directly interact with cell cycle progression and adaptation of metabolism.

Yeast cells possess a number of signaling pathways that enable them to respond to stresses, external nutrients and pheromone (Figure 2).

Although signal transduction in yeast has been studied thoroughly, only a few quantitative models have been published so far [57,58]. A first model of the G protein activation within the pheromone pathway has been presented by Yi and colleagues [6]. This model is based on G protein activities that have been measured using fluorescence resonance energy transfer (FRET). It comprises the production, degradation and activation of the G protein coupled α-receptor (Ste2), the activity cycle of the G protein and its regulation by the G protein regulator (RGS) Sst2 (compare Figure 1).

This model has been adapted and incorporated into a more comprehensive model of the pheromone pathway [52], which includes downstream processes of the activation of Gβγ. As shown in Figure 2, the components of the MAP kinase cascade bind to the scaffold protein Ste5. Binding of Ste5 to Gβγ and the MAP KKKK Ste20 brings Ste20 into the vicinity of Ste11, the MAP KKK, permitting its activation. Furthermore, a cycle of binding, phosphorylation and release of the MAPK Fus3 is considered. Phosphorylated Fus3 triggers the following events including the activation of the transcription factor Ste12, the activation of the cell cycle regulator Far1 and the activation of the RGS Sst2.

The pheromone pathway model includes several feedback loops that help to downregulate the pathway after successful signal transduction. First, the activation of Fus3 leads to a repeated phosphorylation of more Fus3 molecules. Secondly, the activation of Sst2 itself depends on the activation of Fus3. It accelerates the closing of the G protein cycle by enhancing the rate of hydrolysis of Gα-bound GTP. Yi et al. [6] studied strains with either constitutively active or inactive Sst2. Third, the transcription factor Ste12 enhances the expression of the protease Bar1, which is exported, cleaves the α-factor, and thereby counteracts the input signal. Hence, the pathway design ensures the long-term downregulation of the pathway after successful activation of target processes.

The response of yeast to osmotic stress has been described by a model [16] that comprises the high osmolarity glycerol (HOG) pathway, transcriptional regulation, the effect on metabolism and the change in the production of glycerol and an additional model describing regulation of volume and osmotic pressure (see Figure 2). The HOG pathway consists of two input branches, the Sln1 branch and the Sho1 branch (which is not considered in the model). The receptor Sln1 is a membrane protein that senses the osmotic changes. Under normal conditions, it is continuously phosphorylated and transmits its phosphate group to Ypd1, which in turn passes it on to Ssk1. In this way, Ssk1 is kept phosphorylated and inactive. This module consisting of Sln1, Ypd1, and Ssk1 is a so-called phosphorelay system. Upon osmotic stress, phosphorylation of Sln1 is interrupted and Ssk1 switches to a non-phosphorylated, active state. In this form, it triggers the HOG MAP kinase cascade, which involves the redundant proteins Ssk2 and Ssk22 as well as Pbs2 and Hog1. Phosphorylated Hog1 can enter the nucleus and regulate the transcription of a series of genes.

During osmostress, the pathway is downregulated despite sustained external activation. This cellular response could not be explained by modeling the signaling pathway in isolation. It was argued that the turgor pressure instead of the external salt concentration is sensed by the cell. The turgor pressure is partially regulated by glycerol. Active Hog1 activates the expression of genes coding for enzymes that are involved in the production of glycerol.

Model simulations have revealed details of the signaling process, enlightening the role of the glycerol channel Fps1 in glycerol accumulation, and the feedback control exerted by protein phosphatases in the MAP kinase pathway. It turns out that Fps1 is responsible for the immediate control on the internal glycerol concentration, while the stimulated expression of GPD1 and GPP2 and the resulting increased glycerol production preserves a high level of glycerol during growth in high osmolarity. The model implies that the HOG pathway is shut off by glycerol accumulation, cell re-swelling, and turgor increase rather than by enhanced expression of phosphatases. This result has been confirmed by the experimental fact that the pathway can be fully reactivated by a second osmotic stress.

### Jak-Stat pathway

**Figure 3:**
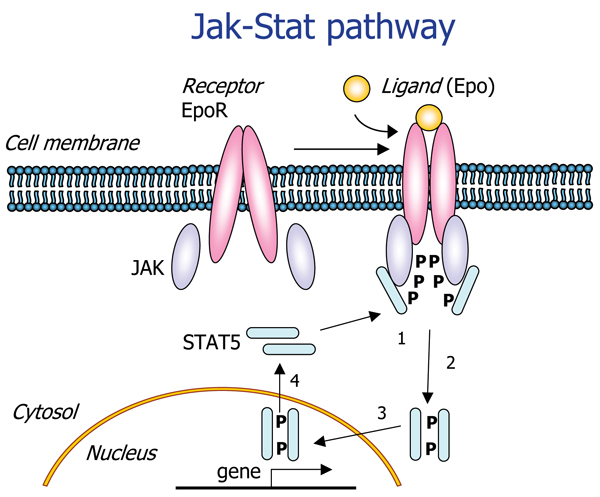
**Jak-Stat Pathways**. Jak-Stat pathways entail the receptor Janus kinase (Jak) and the signal transducer and activator of transcription (Stat). Stat molecules are inactive as monomers, and their activation involves phosphorylation and dimerization. The binding of the ligand (here the hormone Epo) to the receptor (EpoR) results in phosphorylation of Jak and of the cytoplasmatic domain of EpoR. Monomeric Stat5 is recruited to the phosphorylated and thereby activated receptor, EpoR_A_. (1) Upon receptor recruitment, monomeric Stat5 is tyrosine-phosphorylated. It dimerizes (2) and migrates to the nucleus (3), where it binds to the promoter of target genes. After dissociation (4), it is dephosphorylated and exported to the cytoplasm.

Jak-Stat pathways play an important role in regulating immune responses and cellular homeostasis in human health and disease [59,60]. They are activated by cytokines, a large family of extracellular ligands. The family of structurally and functionally conserved proteins involves four Jak-type receptors and seven Stats ("signal transducer and activator of transcription"). Figure 3 shows the structure of a Jak-Stat pathway comprising the Jak-type receptor EpoR and the Stat5.

In a mathematical model of the Jak-Stat pathway presented by Swamaye et al. [7], the dynamics has been described with mass action kinetics for all steps. Stat5 molecules have to reside in the nucleus for a certain time: this has been modeled by a fixed delay in the differential equations. The model showed that recycling of Stat5 molecules is an important detail of the activation cycle and necessary to explain experimental data. The Jak-Stat signaling system in human B-cells was described in a network-based approach [61], and the concept of extreme pathways [62] has been used to study emergent systemic properties of the Jak-Stat signaling network, such as crosstalk between pathways, redundancy of signaling inputs and outputs, and correlated responses.

### EGF receptor signal transduction

The epidermal growth factor (**EGF**) receptor belongs to the tyrosine kinase family of receptors, which regulate cell growth, survival, proliferation, and differentiation. Binding of EGF to the extracellular domain of the EGF receptor induces receptor dimerization and autophosphorylation of intracellular domains. A multitude of signaling proteins are then recruited to the activated receptors through phosphotyrosine-specific recognition motifs. The activation of the small G protein Ras is initiated by two main pathways, one of which depends on Shc, while the other one does not. Active Ras-GTP stimulates the activation of the MAP kinase cascade involving the kinases Raf, MEK, and ERK1/2. Activated ERK phosphorylates and regulates several cellular proteins and nuclear transcription factors

The EGF pathway [63] was modeled to quantify short term signaling and to test the influence of parameter choice and signaling strength. Schoeberl et al. [5] developed a comprehensive model of the EGF receptor-activated MAP-kinase cascade, which contains 94 components. In the model, activated EGR receptor was internalized, and the effect of this feature on signaling and ERK phosphorylation was studied. It could be shown that internalization of receptors contributes little during strong stimulation, but has a strong effect on overall response by retaining signaling capacity at weak stimulation.

### WNT/β-catenin pathway

**Figure 4:**
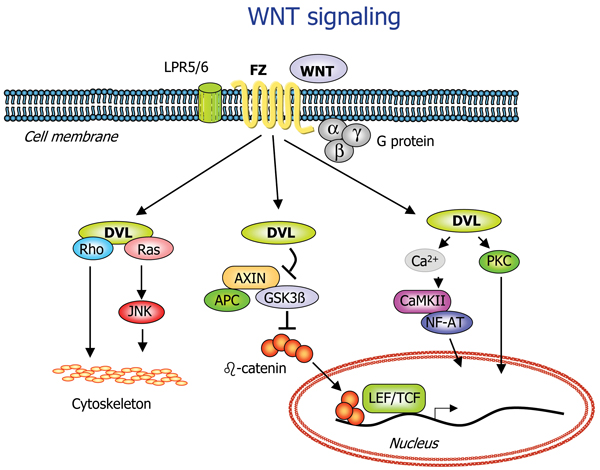
**WNT Pathways**. The extracellular signaling molecule WNT activates three pathways: (1) Early cell fate decisions are controlled via the *canonical pathway *(middle): it comprises the regulation of gene expression by inducing β-catenin-mediated transcriptional activation. Interaction of WNT with the transmembrane receptor frizzled (FZ) activates dishevelled (DVL), which induces the disassembly of a complex consisting of axin, adenomatosis polysis coli (APC), glycogen synthase kinase 3β (GSK3β) and β-catenin. In non-stimulated cells, GSK3β phosphorylates β-catenin, thereby triggering its degradation. Activitation of the pathway effectively increases the levels of β-catenin in the cyctoplasm, which is then translocated to the nucleus. Here it it forms the β-catenin-T-cell specific transcription factor complex that activates the transcription of target genes. (2) In the planar cell polarity pathway (left), FZ functions through G-proteins to activate DVL, which thereupon signals to Rho GTPases (Rho or Rac or both). Activated Ras signals through the c-Jun amino (N)-terminal kinase (JNK). Activation of Rho-GTPases induces changes in the cytoskeleton. In neurons, this pathway is involved in dendritic arborization. (3) In the WNT/calcium pathway (right), activation of DVL activates protein kinase C (PKC) and induces the release of intracellular calcium, which activates a calcium/calmodulin-dependent protein kinase II (CaMKII).

WNT proteins are important mediators of intercellular communication in many cell types, and signaling by members of the WNT family is also crucial for the normal embryonic development of the nervous system. In the nervous system, WNT signaling regulates neuronal connectivity by controlling axon pathfinding, axon remodeling, dendrite morphogenesis, and synapse formation. A recent overview on the WNT pathways can be found in [64] and a schematic representation is given in Figure 4.

WNTs are secreted glycoproteins with an unusual post-translational modification, i. e. palmitoylation at a conserved cysteine, which is essential for their function. The WNT pathway looks as follows: WNT molecules bind to receptors called "Frizzled" (FZ), which are seven-pass transmembrane proteins. The WNT-FZ complex then activates Dishevelled (DVL), a cytoplasmatic scaffold protein that brings together components of the pathway for efficient transduction. Signaling through FZ involves the receptor-related proteins LRP5 or LRP6 and requires G-protein activation. WNT can also signal through the tyrosine kinase-related receptor RYK, which interacts with FZ and DVL. Downstream of DVL, the WNT pathway diverges into at least three branches: the canonical or WNT/β-catenin pathway, the planar cell polarity (PCP) pathway and the WNT/calcium pathway. These pathways again branch to give a pattern of responses.

The WNT/β-catenin pathway has been analyzed in a combined experimental and theoretical study [18] devoted to the understanding of tumor suppression and oncogenecity. The system was modeled by a differential equation model: the kinetics of individual reaction steps are described as simply as possible by either constant rates (for synthesis steps) or mass action kinetics, and conservation relations are considered for DVL, TCF, GSK3β, and APC. The modeling revealed that the two scaffold proteins axin and APC promote the formation of degradation complexes in very different ways. The model also explains the importance of axin degradation in amplifying and sharpening the Wnt signal. Moreover, it shows that the dependence of axin degradation on APC is an essential part of a regulatory loop that prevents the accumulation of β-catenin at low APC concentrations.

Once a system has been modeled, a sensitivity analysis can reveal the effect of parameter changes. In the WNT model, the control of the individual steps over the concentrations of β-catenin and axin has been quantified by means of control coefficients. Strong negative control on the total β-catenin concentration is exerted by the reactions that participate in the assembly of the destruction complex APC/axin/GSK3β, while strong positive control is found in reactions involved in the disassembly of the destruction complex APC/axin/GSK3β and in the dissociation of β-catenin from the destruction complex. The effects of parameter changes on axin were in general opposite to those on β-catenin.

## Signaling pathway models for neurons

Besides their usual role in controlling development (e.g., the growth of axons and dendrites), signaling pathways in nerve cells are also involved in synaptic plasticity, i.e. the variability of the intensity of a signal transmitted through a synapse. Working memory and long-term memory storage in the brain is implemented by short-term and long-term changes in the strength of synapses. An important phenomenon is the spike-time dependent plasticity (STDP): a postsynaptic spike right after transmitter release will strengthen the respective synapses, (long-term potentiation, LTP), while a spike occurring before activation will weaken them (long-term depression, LTD). Practically, the synapse strength is tuned by modification, insertion and removal of receptors, and by remodeling of the actin cytoskeleton in the spine. These processes are regulated by a plethora of biochemical signaling pathways in the postsynaptic spines of excitatory synapses, which are controlled by the activity of the synapse and by hormonal influences. Two main types of signal transmission contribute to synaptic plasticity: (i) cascades of covalent modifications of existing synaptic proteins (typically protein phosphorylation), resulting in altered synaptic function and (ii) concentration changes of second messengers, which regulate gene transcription and interfere with the protein modification cascades. One of them is the calcium ion, Ca^2+^, which plays an important role in spike-time dependent plasticity: The precise relative timing of transmitter release and postsynaptic spike is sensed by the N-methyl-D-aspartate-type glutamate receptor (NMDAR). This receptor allows influx of Ca^2+ ^into the spine when it binds glutamate and the membrane is depolarized at the same time.

### Calcium-calmodulin models

Bursts of activity in neurons are generated by voltage- and time-dependent ion fluxes, which result from a dynamic interplay among ion channels, second messenger pathways and intracellular Ca^2+ ^concentrations. This interplay is tuned by neuromodulators and synaptic inputs. This complex intrinsic and extrinsic modulation of pacemaker activity exerts a dynamic effect on the activity of the signaling network [65].

**Figure 5:**
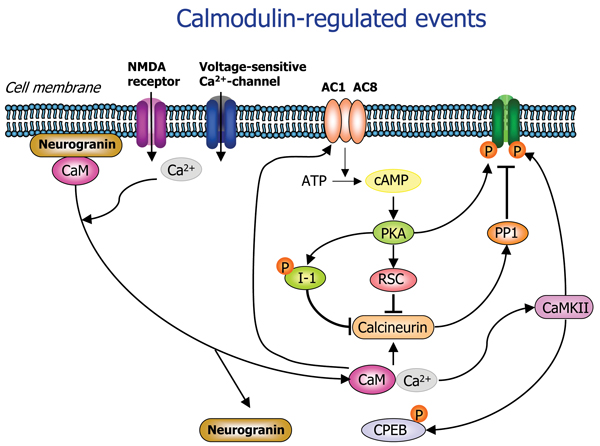
**Calcium-calmodulin network**. Ca^2+^-signaling is mediated through several Ca^2+^-binding proteins, including calmodulin (CaM) and protein kinase C (PKC). The activity of *N*-methyl-d-aspartate (NMDA) receptors or voltage-sensitive Ca^2+ ^channels leads to an increase in intracellular Ca^2+^, which triggers a release of calmodulin that was previously bound to neuromodulin or neurogranin. Depending on Ca^2+^, CaM modulates the activity of several key signaling molecules that are crucial for synaptic plasticity including adenylyl cyclases (AC), protein kinases, calcineurin, nitric oxide synthase, Ca^2+^-channels, ATP-dependent Ca^2+^-pumps, and the CaM-dependent protein kinases (CaMKII). CaM has four Ca^2+ ^binding sites and in the presence of CaM binding protein, it shows heterotropic positive cooperativity for Ca^2+^. This enables that CaM-regulated AC and cyclic nucleotide phosphodiesterases have different Ca^2+^-sensitivities, and that CaM-stimulated phosphatase calcineurin has greater sensitivity to Ca^2+ ^than CaMKII.

Calcium spikes are thought to encode information mainly by their frequency, not by their amplitude. It is an interesting question how non-excitable cells decode these signals into a frequency-dependent cellular response. This question was tackled by Goldbeter and coworkers [66-68] by computational modeling. Calcium interacts with the calcium/calmodulin-dependent protein kinase type II (CaM kinase II), which is a ubiquitous, multifunctional enzyme. Protein phosphorylation was suggested [66,69] as a possible mechanism for frequency decoding of Ca^2+ ^oscillations. A general model for frequency decoding of Ca^2+ ^oscillations [66] takes into account a phosphorylation-dephosphorylation cycle with a Ca^2+^-activated kinase and a Ca^2+^-independent phosphatase (Figure 5). The role of CaMKII in decoding Ca^2+ ^oscillations has been studied by theoretical models, which have predicted that the level of activity of CaMKII increases with the frequency of Ca^2+ ^oscillations due to a sort of time integration of the oscillatory signal [69-73].

### Combined pathway models for signaling in neuronal cells

In addition to synaptically mediated signals that are based on changes in membrane potential, neurons generate and receive many types of signals that involve biochemical pathways, some of which are independent of voltage. Interactions between such biochemical pathways involving positive and negative feedback loops lead to emergent system properties, i.e. properties that rely not only on the individual properties of proteins but on their connections, most notably bistability and oscillations. Due to this interdependence, the involvement of biochemical pathways in information processing increases the computational and integrative capacity of a neuron.

**Figure 6:**
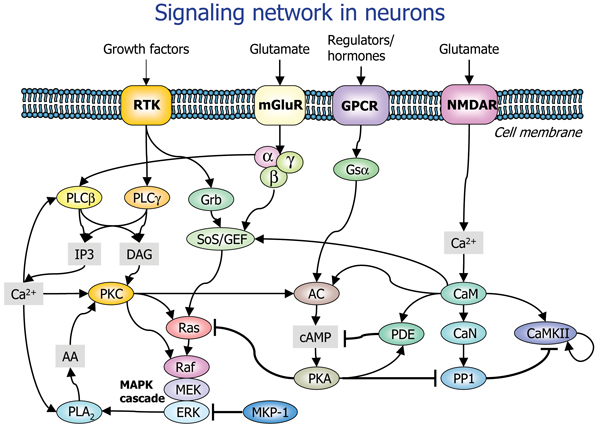
**Signaling network in neurons **Schematic overview of signaling pathway modules in neuronal cells (redrawn from Bhalla [82]). Abbreviations: RTK: receptor tyrosine kinase; mGluR: metabotropic glutamate recptor; GPCR: G-protein coupled receptor; NMDAR: *N*-methyl D-aspartate Receptor; Gq: G-protein type q; Gsα: G-protein type s; PLCβ: phospholipase C β; PLCγ: phospholipase C γ; IP3: inositol trisphosphate; DAG: diacylglycerol; Sos/GEF: Son of Sevenless/guanine nucleotide exchange factor; Ca^2+ ^: Calcium; PKC: protein kinase C; AC: adenylyl cyclase; PDE: phosphodiesterase; CaM: calmodulin; CaMKII: calcium calmodulin kinase type II; cAMP: cyclic adenosine monophosphate; CaN: calcineurin; AA: arachidonic acid; PLA_2_: phospholipase A2; MAPK: mitogen activated protein kinase; MKP-1: MAP-Kinase phosphatase type 1; PKA: protein kinase A; PP1: protein phosphatase type 1.

Hence an important issue in modeling of signaling systems is the integration of pathways. In a series of excellent papers, Bhalla and coworkers developed computational models for different signaling pathways in neurons. [e.g. [17], e.g. [74-76]]. They also integrated several individual pathways into networks. First, individual pathways were modeled by sets of differential equations and their behavior was tested against experimental data. In the models, the individual reaction steps were described by mass action kinetics, while enzyme-catalyzed reactions were described by a two-step scheme in which each individual step is covered by mass action. The aim of the study was to examine emergent properties of the coupled signaling network: two or three pathway models were iteratively combined to more complex models and their behavior was tested again against published data. Figure 6 shows the pathways and how they were linked.

Besides aiming at agreement with experimental data, the combined models could explain several regulatory features of the network: The downregulation of the MAPK activity after initial stimulation (by a constant EGF stimulation) stems from a combined effect of EGF receptor internalization, MAPK phosphorylation and inactivation of SoS. Moreover, a positive feedback loop contributes to bistable behavior, i.e. depending on the value of the initial concentrations of compounds or on the choice of stimulation scenarios, different steady states of the system will be reached. Specifically, the impact of amplitude and duration of the EGF stimulation on MAPK activation was studied. Weak or short-term stimulation results in transient activation, while only strong long-term activation provokes sustained MAPK activation. This can be tested in time course plots, where concentrations are plotted versus time. More elegantly, bistability is analysed using a phase plane plot showing the nullclines of the two players in the feedback loop, PKC and MAPK. Two of the three steady states are stable, pointing to bistability. The unstable steady state marks the threshold between both types of stimulation (or, in mathematical terms, marks the separatrix distinguishing the basins of attraction for the two stable steady states).

In [17], Bhalla et al. also tested the influence of phosphatases on the MAPK activation. Simulating a phosphatase that is active in a defined period at a defined level, again only strong, long lasting action of the phosphatase was found to downregulate MAPK, while weak or short phosphatase activity was not sufficient. Hence, it was claimed that small fluctuations will keep the output of such systems unaffected.

It was found that the integrated network shows emergent properties that the individual pathways do not possess, like extended signal duration, activation of feedback loops, thresholds for biological effects, or a multitude of signal outputs

## Evolutionary design and input signals of signaling pathways

The application of optimality principles can shed light on the design of biochemical networks and on their evolutionary development. Different processes in molecular biology have been studied in terms of optimality, such as the distribution of phosphorylation steps in glycolysis based on flux maximization or the temporal order of gene expression in biosynthetic pathways based on minimal transition time [77-81].

From an engineer's point of view, signaling pathways have evolved to perform tasks like signal processing, filtering, pattern recognition, and discrimination of time series. It can be insightful to ask: "How would an engineer have designed a pathway with a given task?", but this question often seems to be perpendicular to biology. First, biological systems have evolved step by step, by adding little modifications to the composition already acquired. Secondly, signaling pathways usually do not execute a single stereotypic task, but have to ensure survival in an unstable environment with a multitude of demands. For instance, one may doubt that biological signaling pathways resemble simple on/off switches or tunable elements designed by engineers: Their architecture is much more diverse and flexible. Papin et al. [3] have pointed out that, due to alternative splicing and posttranslational modifications, the potential number of different signaling proteins can be enormous.

Understanding the evolutionary design of signaling pathways work is also hampered by an experimental fact: important information about the physical design of pathways is obtained from analysis of mutant, artificially altered organisms or strong stress signals. In *in vivo *experiments, cells are typically stressed with a high amount of a specific stressor applied for long times. For example, the pheromone pathway in yeast cells responds to the pheromone α-factor by changes of molecular activation states, which should occur within a few minutes. Measurements every 30 min (as frequently presented) would miss relevant events in pathway activation and downregulation. The subsequent directed formation of a shmoo tip necessitates gradients of the external stimulus. This behavior will certainly not be observable if yeast cells are put for two hours in an environment with an isotropic distribution of α-factor.

Laboratory conditions, in general, do not mirror the natural environment that cells have faced during evolution and for which they have evolved the specific pathway. Therefore, the design of the pathways will not appear optimal in the experimental setting. Moreover, if the experiments do not reveal some important modes of behavior, they provide less useful information for fitting and validating the models.

Obviously, it is easier to formulate demands from the perspective of a dry lab than fulfilling them in a wet lab. Nevertheless, it seems to be necessary to test more situations that mimic realistic stress conditions in order to learn which mechanisms cells have evolved to cope with their normal environment. The need to understand signal transduction and cellular regulation and to apply the findings in biotechnology and health care will focus future research to conditions as close as possible to natural environment. Especially under the umbrella of systems biology, experimentalists and modelers from different disciplines work closely together to exchange experience, knowledge and awareness of the requirements of each other's approach.

## Authors' contributions

Both authors contributed equally to this work.
